# Acquired bedaquiline resistance in Karakalpakstan, Uzbekistan

**DOI:** 10.5588/ijtld.21.0631

**Published:** 2022-07-01

**Authors:** P. Nair, T. Hasan, K. K. Zaw, S. Allamuratova, A. Ismailov, P. Mendonca, Z. Bekbaev, N. Parpieva, J. Singh, N. Sitali, E. Bermudez-Aza, A. Sinha

**Affiliations:** 1 Médecins Sans Frontières (MSF) Holland, Amsterdam; 2 Republican Center of Tuberculosis and Pulmonology, Nukus, Uzbekistan; 3 Republican Specialized Scientific and Practical Medical Center of Tuberculosis and Pulmonology, Tashkent, Uzbekistan; 4 MSF, Berlin, Germany; 5 MSF, Amsterdam, The Netherlands; 6 MSF, London, UK

**Keywords:** MDR-TB, XDR-TB, antibiotic resistance, drug resistance

## Abstract

**BACKGROUND:**

The WHO recommends the use of bedaquiline (BDQ) in longer, as well as shorter, multi-drug-resistant TB (MDR-TB) treatment regimens. However, resistance to this new drug is now emerging. We aimed to describe the characteristics of patients in Karakalpakstan, Uzbekistan, who were treated for MDR-TB and acquired BDQ resistance during treatment.

**METHODS:**

We performed a retrospective study of routinely collected data for patients treated for MDR-TB in Karakalpakstan between January 2015 and December 2020. We included patients on BDQ-containing regimens with baseline susceptibility to BDQ who developed BDQ resistance at any point after treatment initiation. Patients resistant to BDQ at baseline or with no confirmed susceptibility to BDQ at baseline were excluded.

**RESULTS:**

Of the 523 patients who received BDQ-containing regimens during the study period, BDQ resistance was detected in 31 patients (5.9%); 20 patients were excluded—16 with no prior confirmation of BDQ susceptibility and 4 who were resistant at baseline. Eleven patients with acquired BDQ resistance were identified. We discuss demographic variables, resistance profiles, treatment-related variables and risk factors for unfavourable outcomes for these patients.

**CONCLUSION:**

Our programmatic data demonstrated the acquisition of BDQ resistance during or subsequent to receiving a BDQ-containing regimen in a patient cohort from Uzbekistan. We highlight the need for individualised treatment regimens with optimised clinical and laboratory follow up to prevent resistance acquisition.

## Abstract (fr)

**CONTEXTE:**

L’OMS recommande l’utilisation de la bédaquiline (BDQ) dans le cadre des schémas thérapeutiques longs et courts de la TB multirésistante (MDR-TB). Toutefois, des résistances à ce nouveau médicament commencent à apparaître. Notre objectif était de décrire les caractéristiques des patients du Karakalpakistan, Ouzbékistan, traités pour MDR-TB et ayantacquisunerésistance à la BDQencoursde traitement.

**MÉTHODES:**

Nous avons réalisé une étude rétrospective des données recueillies en routine auprès des patients traités pour MDR-TB au Karakalpakistan de janvier 2015 à décembre 2020. Nous avons inclus les patients sous schémas à base de BDQ avec sensibilité à la BDQ à l’inclusion et ayant développé une résistance à la BDQ à tout moment après l’instauration du traitement. Les patients présentant une résistance à la BDQ à l’inclusion ou sans sensibilité confirmée à la BQD à l’inclusion ont été exclus.

**RÉSULTATS:**

Un total de 523 patients ont reçu des schémas à base de BDQ pendant la période de l’étude. Une résistance à la BDQ a été détectée chez 31 patients (5,9%). Vingt patients ont été exclus : 16 sans confirmation antérieure de sensibilité à la BDQ et quatre présentant une résistance à l’inclusion. Onze patients avec résistance acquise à la BDQ ont été identifiés. Nous avons analysé les variables démographiques, les profils de résistance, les variables liées au traitement et les facteurs de risque de résultats défavorables pour ces patients.

**CONCLUSION:**

Nos données programmatiques ont démontré l’acquisition d’une résistance à la BDQ pendant ou après un traitement à base de BDQ au sein d’une cohorte de patients d’Ouzbékistan. Des schémas thérapeutiques personnalisés sont nécessaires, avec suivi biologique et clinique optimisé, pour prévenir l’acquisition de cette résistance.

Drug-resistant TB (DR-TB) is a growing public health challenge, with around half a million cases of multidrug-resistant (MDR) or rifampicin-resistant (RR) TB estimated in 2019.^1^ As the body of research evolves, especially with the introduction of new and repurposed drugs, MDR-TB treatment is being adapted. In its most recent DR-TB treatment guidelines,^2^ the WHO therefore recommends MDR-TB to be treated with a regimen composed of at least four drugs during an 18 to 20-month period, based on baseline resistance profile. This regimen includes three drugs categorised as Group A: a fluoroquinolone (FQ; e.g., levofloxacin/moxifloxacin), bedaquiline (BDQ) and linezolid (LZD), plus one additional drug. The standardised shorter regimen with BDQ (duration: 9–12 months) is an alternate for treatment-naïve patients. Hence, the majority of individuals diagnosed with MDR-TB will be on a BDQ-based regimen. While the inclusion of BDQ has enabled the shortening of regimens, there is a risk – as for any antimicrobial agent – that resistance will evolve with such changes.

A recently published WHO expert consultation meeting report for the definition for extensively drug-resistant TB (XDR-TB) has highlighted the importance of testing for BDQ susceptibility.^3^ XDR-TB is now defined as TB caused by strains that fulfil the definition of MDR/RR-TB,^3^ as well as being resistant to any FQ, and at least one additional Group A drug. Although drug susceptibility testing (DST) of FQs is now widely available, access to DST for other Group A drugs, i.e., BDQ and LZD, is very limited and unavailable in many high MDR-TB burden countries.

There is little published data on prevalence of BDQ resistance and development of resistance during treatment. A phenotypic drug susceptibility test (pDST) study on samples from 124 patients in Germany showed 7 of 124 (5.6%) isolates with a minimum inhibitory concentration (MIC) for BDQ greater than the critical concentration (CC), and resistance developing in four of these patients despite individualised regimens.^4^ A laboratory study carried out in Russia documented 126 isolates from patients receiving BDQ for more than 90 days having acquired mutations known to confer reduced susceptibility to BDQ.^5^ Development of resistance to BDQ during treatment has also been reported in a study in Pakistan.^6^ Of 30 patients with consistent culture positivity on treatment, six patients with baseline BDQ sensitivity demonstrated increased MIC during therapy; five of six patients eventually developed an MIC greater than the CC.

Uzbekistan, a Central Asian country with a population of 33 million, had 2,060 laboratory-confirmed cases of RR/MDR-TB in 2019; 12% of new, and 22% of retreatment cases of TB, were estimated to have RR/MDR-TB.^7^ Since 2003, Médecins Sans Frontières (MSF) has worked in close collaboration with the Ministry of Health (MoH) of Karakalpakstan, an autonomous republic in the northwest of Uzbekistan, to strengthen TB surveillance, prevention and care.^8^ Since 2015, MSF has supported the MoH in the programmatic implementation of BDQ-based regimens, followed by delamanid in 2018.

The understanding of the scale and risks of BDQ resistance is limited, both globally and in Uzbekistan. We aimed to describe the characteristics of patients who were infected with strains confirmed as susceptible to BDQ, but subsequently acquired resistance, during the course of treatment in Karakalpakstan, Uzbekistan.

## METHODS

**Figure 1:**
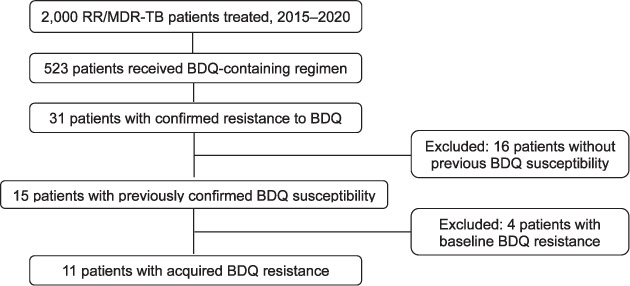
Study population. RR/MDR-TB = rifampicin/multidrug-resistant TB; BDQ = bedaquiline.

This study used routine programme data collected from standardised patient forms and encoded in the MSF programme database. Data from records for all RR/MDR-TB patients treated in 2015–2020 by the MSF-supported project in Karakalpakstan were included for evidence of treatment with BDQ-containing regimens and confirmed resistance to BDQ. Figure 1 presents the selection process for the study population.

Resistance to BDQ was confirmed using pDST for BDQ, and was performed with the proportion method, using the BACTEC Mycobacterial Growth Indicator Tube (MGIT) 960 (BD, Franklin Lakes, NJ, USA) at a CC of 1 mg/L. The process passes annual checks of external quality assurance by the Supranational Laboratory, Gauting, Germany. pDST for BDQ was introduced in the MSF-supported TB project in Karakalpakstan in September 2019, and was initially performed on samples from patients on MDR/RR-TB treatment who had failed to culture convert and on baseline previous samples that had been stored at an earlier date. pDST for BDQ was subsequently incorporated into routine testing algorithms for RR/MDR-TB patients in January 2020. The review was conceptualised as a case-control study, but given the small sample size, it was modified to a case series.

Variables extracted for identified patients confirmed to have developed BDQ resistance at any point after initiation of treatment were age, sex, drugs administered during previous treatment(s), past drug-susceptible treatment, past drug-resistant (DR) treatment, outcome of last treatment episode and past clofazimine (CFZ) exposure. Additionally, the following data were extracted pertaining to the time of initiation of the BDQ-containing regimen: time on treatment before starting BDQ, likely effective drugs^2^ (as defined by the WHO) at time of BDQ start, all drugs in the BDQ-containing regimen and resistance profile.

Time from BDQ start to resistance was calculated in months. BDQ resistance was categorised as either detected during, or after treatment. We also extracted data for the number of days between cessation of treatment and detection of resistance. Estimation of missed days was calculated according to records, and in addition, details of any adverse events, culture conversion outcomes and final treatment outcomes, were obtained.

Finally, the following tests were included in the analysis: hepatitis B surface antigen (HBsAg), anti-hepatitis C virus antibody (anti-HCV Ab), HIV antibody and type 2 diabetes mellitus (blood glucose). Baseline characteristics were described using frequencies and percentages for categorical variables, and medians with interquartile ranges (IQRs) for continuous variables.

As the study fulfilled the exemption criteria set by the MSF independent Ethical Review Board (ERB) for a posteriori analyses of routinely collected clinical data, it did not require MSF ERB review.

## RESULTS

In Karakalpakstan, Uzbekistan, 2,000 patients were treated for DR-TB from January 2015 to December 2020. Of this cohort, 523 received a BDQ-containing regimen. BDQ resistance was detected in 31 of these patients. Subsequently, 20 patients were excluded, 16 because of no previous BDQ susceptibility and 4 because they were resistant to BDQ at baseline. The final study population comprised 11 patients who were confirmed to have developed BDQ resistance at any point after initiation of treatment (Figure 1).

Table 1 gives an overview of demographic data, past treatment and relevant clinical data at initiation of the BDQ-containing regimen. Factors related to past TB treatment are the number of TB treatment episodes and drugs taken prior to the current treatment episode. Treatment outcomes refers to previous treatment course. Clinical data include the resistance profile, number of likely effective drugs and composition of the BDQ-containing regimen and comorbidities known to cause unfavourable outcomes. Comorbidities analysed were hepatitis C, i.e., anti-HCV Ab, HIV status, hepatitis B (HBsAg) and diabetes mellitus; specific comorbidity is mentioned in the table only if detected for the concerned patient.

**Table 1:** Patient data at time of BDQ initiation

Study ID	Age at initiation years	Sex	History of past treatment (DS/DR)	Outcome of last treatment episode	Past CFZ exposure	Resistance profile	BDQ-containing regimen*	Likely effective drugs	Comorbidity
A	32	M	0 DS/2 DR	LTFU	Yes	Pre-XDR + SLI-R	BDQ, LZD, IPM/CLN, MFX, PTH, CS	3	
B	58	M	1 DS	Treatment completed	No	MDR + SLI-R	BDQ, LZD, IPM/CLN, PTH, MFX	4	
C	65	M	1 DS/DR	Failure	No	Pre-XDR + SLI-R	BDQ, CFZ, LZD, IPM/CLN, MFX, AmxClv	4	
D	42	M	1 DR	Failure	No	Pre-XDR + SLI-R	BDQ, CFZ, LZD, PTH, PZA, CPM, MFX	4	Yes; DM; HBsAg-positive
E	32	M	1 DR	Unknown	No	Pre-XDR	BDQ, CFZ, LZD, CPM, PTH, PZA, MFX	5	
F	24	M	1 DS/1 DR	LTFU	Yes	Pre-XDR + SLI-R	BDQ, LZD, IPM/CLN, MFX, PTH	3	
G	49	F	None	None	Yes	Pre-XDR	BDQ, CFZ, LZD, CS, KM	5	Yes; DM
H	37	M	3 DR	LTFU	Yes	MDR + SLI-R	BDQ, LZD, PZA, MFX, CFZ	2	
I	26		2 DR	LTFU	Yes	Pre-XDR	BDQ, LZD, IPM/CLN, CM, PTH, CS, PAS	3	
J	31	F	1 DS/2 DR	Cure	Yes	Pre-XDR	DLM, IPM/CLN, BDQ, AMK, CFZ, LZD	1	
K	18	M	None	None	No	Pre-XDR	BDQ, DLM, CFZ, LZD, CS	5	

* Likely effective drugs underlined.

BDQ = bedaquiline; DS = drug-susceptible; DR = drug-resistant; CFZ = clofazamine; M = male; LTFU = lost to follow-up; XDR = extensively drug-resistant; SLI = second-line injectables; R = rifampicin; LZD = linezolid; IPM/CLN = imipenem/cilastatin; MFX = moxifloxacin; PTH = prothionamide; F = female; CS = cycloserine; MDR = multidrug-resistant; AmxClv = amoxicillin-clavalunate; CPM = capreomycin; PAS = para-aminosalicylic acid; AMK = amikacin.

**Figure 2:**
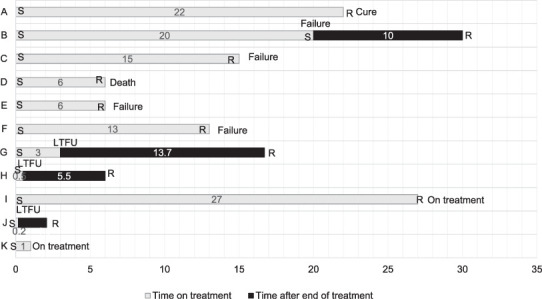
BDQ resistance detection timeline. Grey bars represent time on treatment and black bars the time after stopping treatment. The end point of the timeline is development of resistance. The timing of culture conversion (if applicable) and outcome is mentioned in the figure. S = susceptible; R = resistant; BDQ = bedaquiline.

Details regarding acquisition of BDQ resistance are presented in Table 2. We highlight culture conversion, if applicable; adherence (number of missed days between BDQ start and detection of resistance, and total number of days without TB treatment between BDQ start and detection of resistance); adverse events; time on BDQ-containing treatment when BDQ resistance was identified; treatment duration; and outcome. The median time from BDQ start to resistance was 12 months (IQR 5.5–18.5) (Figure 2).

**Table 2:** BDQ-containing treatment data

Study ID	Time from BDQ start to resistance Months	BDQ resistance detected during or after treatment	Time after stopping treatment when resistance detected days	Missed days*	Adverse events	Unplanned treatment changes	Culture conversion	Treatment outcome
A	22	During	NA	5	No	10	Yes (M14)	Cure
B	30	After*	290	383	Yes, nausea, vomiting	12	No	Failure
C	15	During	NA	41	Yes, nausea	13	No	Failure
D	6	During	NA	5	Yes, nausea	6	No	Death
E	6	During	NA	24	No	3	No	Failure
F	14	During	NA	8	Yes, nausea	12	No	Failure
G	12	After	417	417	Yes, anaemia	2	No	LTFU
H	5	After*	158	158	No	0	No	LTFU
I	27	During	NA	14	Yes, nausea	11	No	On treatment
J	2	After*	58	58	Yes, fever temporally related to IPM/CLN	0	No	LTFU
K	1 month (27 days)	During	NA	5	No	0	Yes (M1)	On treatment

* These patients were susceptible to BDQ shortly before stopping treatment. Resistance was detected at a later date and after stopping treatment. BDQ = bedaquiline; NA = not applicable; LTFU = loss to follow-up; IPM/CLN = Imipenem/cilastatin.

## DISCUSSION

We present the findings in a cohort of 11 patients treated for MDR-TB in Karakalpakstan, Uzbekistan, who acquired BDQ resistance after the exposure to BDQ therapy as part of a TB treatment regimen. Resistance developed both early and late during the course of treatment, and after completion of course of treatment with a BDQ-based regimen; however, exact time is uncertain as monthly pDST is not carried out for culture-positive casesin this programmatic setting.

We draw parallels with the conclusions of a South African cohort study^9^ in people living with HIV with DR-TB,^2^ which identified resistance to FQs or second-line injectables, and past TB treatment, as factors to be considered. A further study from Abhkhazia, Georgia, also suggests that unfavourable outcome from previous treatment later leads to increased resistance to anti-TB drugs,^10^ as this may reduce the number of likely effective drugs, compromising the efficacy of the background regimen.

In our case series, 6 of 11 (54.5%) patients had four or more likely effective drugs as recommended by the WHO^2^ in their TB treatment regimen. However, despite this, BDQ resistance seems to be a predictor of adverse treatment outcome. Furthermore, two patients (H and J) rapidly developed BDQ resistance (in <6 months) due to the lack of likely effective drugs in their treatment regimen. As proposed in a similar study in Pakistan, it would also be advisable to consider the complementary activity of these drugs to prevent acquisition of BDQ resistance.^11^

A total of 6 of 11 (54.5%) patients were exposed to CFZ during past treatment, of which 1 (Patient J) of 11 (9.1%) was exposed to both BDQ and CFZ during the previous treatment episode. Both of Patient J’s samples were re-checked in the laboratory. We speculate that the patient had heteroresistance/low-level resistance to BDQ arising from previous exposure to both BDQ and CFZ, which was unmasked in the second sample.

As baseline pDST for CFZ was not available for most patients, this was not mentioned in our study. Cross-resistance between BDQ and CFZ due to the Rv0678 mutation has been highlighted in many studies.^4^,^6^,^9^ A South African cohort study demonstrated the presence of the Rv0678 mutation in 92 sequenced isolates.^9^ In that study, 5 of 92 (5.4%) patients were treatment-naïve for BDQ, with MICs below the CC; Rv0678 mutations emerged during treatment in a further 5 of 87 (5.7%) patients with samples now showing a more than eight-fold increase in MIC. A recommendation about carrying out pDST at MICs lower than the CC was made in that study.^9^ We suggest further investigation with a larger cohort in Uzbekistan and the use of next-generation sequencing to determine the presence of Rv0678 mutations. It would also be worthwhile to explore carrying out baseline and sequential pDST for BDQ at lower MICs than the WHO-recommended CC, as this could unmask low-level resistance to BDQ.

Adverse events are known to cause both patient-and physician-derived treatment interruption,^12^ whether this is based on the physician’s or the patient’s decision. Seven of 11 patients (63.6%) had side effects in our study while on TB treatment, the most common being nausea, observed in five of seven patients. These adverse events may have contributed to the large number of missed days, and we hypothesise that this may be a factor in the development of BDQ resistance. We therefore emphasise the need for close follow-up and early management of adverse events in order to avert frequent changes in treatment regimen or interruption of treatment.

Treatment was missed by nine of 11 (81.8%) patients during their time on a BDQ-containing regimen. Missed days were calculated as time off drugs (days) from the treatment initiation until resistance detection. This means that the time (days) between stopping treatment and detection of resistance were also counted, as applicable, as missed days. It is important to note that in four of 11 (36.3%) of these patients (B, failure; G, H and J all lost to follow-up), BDQ resistance was detected after stopping treatment, although three (B, H and J) of these four patients tested susceptible to BDQ shortly before stopping treatment. For Patient G, the BDQ-resistant result was sub-cultured from a stored sample which was the baseline sample for a new regimen. BDQ resistance in Patients B, H and J was detected as part of follow-up monitoring; however, these patients were not started on new regimens due to the lack of likely effective drugs (Patient B) and patient refusal (Patients H and J). This is similar to a case report from South Africa,^13^ and is important given the long terminal half-life of BDQ. In the case of failure resulting from loss to follow-up, patients are theoretically on monotherapy with BDQ for 5.5 months after the end of treatment.

It is important to note that while national guidelines follow the WHO definition of outcomes, implementation by the consilium (weekly medical meeting of local TB specialists) could vary. Patient A was declared cured despite a late culture conversion at 17 months.^2^ Furthermore, Patient I remains on treatment despite being consistently culture-positive at the time of this review (December 2020, Month 30 of treatment) but treatment was continued. Timely declaration of outcome, including failure as in Patients C and E, is advisable to prevent side effects, given the minimal added benefits of continuing therapy.

The result we find most worrying was that of Patient K who had no history of treatment and seemed to acquire BDQ resistance in 1 month. The baseline result was rechecked and confirmed to be accurate. This suggests either the unmasking by selection pressure of the drugs of BDQ heteroresistance,^14^ or as discussed earlier low-level resistance below the CC.^9^

The study relies on programmatic data and therefore reflects real-world settings. However, a major limitation is that BDQ pDST could not be performed at baseline for all 31 patients in whom BDQ resistance was detected but only for those for whom samples were available. The acquisition of BDQ resistance during treatment in 11 of 31 cases is thus possibly an underestimate. Also, as BDQ resistance was not tested monthly (especially after outcome), the timing of resistance acquisition is uncertain. The small sample size and the lack of comparison data made it impossible to assess predictors of BDQ resistance.

## CONCLUSION

This study focuses on a subset of XDR-TB patients,^3^ specifically those resistant to BDQ. It raises an important point regarding development of BDQ resistance after stopping treatment when an unfavourable outcome occurs. Our findings reinforce the importance of monitoring for acquired drug resistance during TB therapy.

The development of drug resistance has significant patient and public health implications. We suggest that patients must be identified at time of initiation as having significant risk of developing BDQ resistance and regimens strengthened accordingly to prevent resistance amplification. Due to the small sample size, it was not possible to assess predictors of BDQ resistance. However, the hypotheses developed can be used as the basis for future cohort studies. We strongly recommend that individualised treatment regimens, combined with close follow up and management of side effects, be implemented in order to ensure the achievement of favourable outcomes and the prevention of acquired resistance to BDQ.
